# How do stressful life events affect medical students’ academic adjustment? Parallel mediating mechanisms of anxiety and depression

**DOI:** 10.1186/s12909-024-05601-0

**Published:** 2024-06-04

**Authors:** Hui-Bing Guo, Chen Qiu

**Affiliations:** 1 Student Affairs Department, West China School of Medicine, West China Hospital, Sichuan University, No. 37, Guoxue Alley, Wuhou District, Chengdu, Sichuan China; 2 West China School of Nursing, Sichuan University, Chengdu, China

**Keywords:** Stressful life events, Academic adjustment, Depression, Anxiety, Medical students

## Abstract

**Background:**

Medical students frequently face challenges in academic adjustment, necessitating effective support and intervention. This study aimed to investigate the impact of stressful life events on medical students’ academic adjustment, focusing on the mediating roles of depression and anxiety. It also differentiated the impacts between at-risk students (those with academic failures) and their peers respectively.

**Methods:**

This case-control study involved 320 at-risk medical students and 800 other students from a university in western China. Participants anonymously completed the scales of stressful life events, depression, anxiety, and academic adjustment. T-tests, ANOVA, Pearson correlation, and structural equation modeling were employed for statistical analysis.

**Results:**

Depression and anxiety were significantly more prevalent in at-risk students (46.8% and 46.1%, respectively) than in controls (34.0% and 40.3%, respectively). Notably, at-risk students had poorer academic adjustment (*t* = 5.43, *p* < 0.001). The structural equation modeling had good fit indices and the results indicated that depression and anxiety fully mediated the effects of stressful life events on academic adjustment. For at-risk students, stressful life events significantly decreased academic adjustment through increased depression and anxiety (*p* < 0.001). Conversely, anxiety had a positive effect on academic adjustment in other students.

**Conclusion:**

Targeted interventions focusing on depression and anxiety could reduce the negative impact of stressful life events on medical students’ academic adjustment. However, educators have to distinguish the differences between at-risk students and others.

## Background

 Academic adjustment is a fundamental element of educational success, encompassing the process of adapting to new educational environments [3, 41], This dynamic process is not confined to the initial stages of freshman enrollment but continues throughout the college journey [12, 35]. Previous studies have consistently shown that academic adjustment exerts a substantial impact on students’ academic performance, with far-reaching implications for their employment and career success [36, 37]. Conversely, academic maladjustment can precipitate severe educational setbacks, such as failing final exams, downgrading, or dropping out of university [2].

Medical students, in particular, face exceptional challenges due to their demanding course loads, frequent assessments, and increased professional expectations [11, 28]. According to Winston et al. [42], students who fail final exams can be defined as at-risk students. They encounter significant challenges in adapting to higher education and are more likely to drop out or dismissal. Traditional teaching methods focused solely on passing the next test have proven ineffective for this group [7]. Therefore, it is imperative to implement alternative remedial measures tailored to enhance academic adjustment [34]. However, current interventions often lack a strong empirical and theoretical foundation. This study seeks to address this research gap by investigating the underlying mechanisms of academic adjustment among medical students, especially at-risk students.

Studies have demonstrated the significant impact of stressful life events on adolescents’ life trajectories, specifically on their academic development [30]. Dupéré et al. [10] proposed that severe stressful life events may be the determining factor in student dropout. In previous studies, medical students have reported many stressful life events, including illness and bereavement, financial stress, interpersonal interactions, and high workloads [18, 20, 29]. Hojat et al. [17] indicated in a longitudinal study of medical students that stressful life events were positively correlated with academic burnout and negatively correlated with academic performance. Furthermore, evidence suggests that students who experienced more stressful life events reported poorer school adjustment [1, 25]. However, the mediating process of stressful life events’ influence on medical students’ academic adjustment is still being determined.

According to stress coping theory, stressful life events induce negative emotional responses, and individuals under stress have difficulty coping, which in turn leads to psychological or academic problems [22, 40]. This model is divided into two stages. Initially, individuals experienced stressful life events and produced emotional reactions, most commonly depression and anxiety [21, 39]. Depression and anxiety were also recognized as prevalent emotional challenges among medical students [28]. Subsequently, these negative emotional reactions contribute to individuals’ maladjustment. Supporting this, Cortés-Denia et al. [8] found that depression and anxiety directly diminish academic performance and adjustment among adolescents. Furthermore, Ji et al. [20] demonstrated that stressful life events could affect college students’ academic engagement through depression. Recent studies further suggest that anxiety and depression may serve as mediating variables in predicting college adjustment [6]. Based on these evidences, we hypothesized that stressful life events reduce medical students’ academic adjustment through the mediating effects of depression and anxiety.

In addition, guided by the vulnerability-stress model, stressful life events and individual sensitivities add together in some way to produce outcomes [15]. It is also the case that stressful life events might strengthen individuals’ vulnerability [19]. Therefore, we can assume that at-risk students, already impacted by academic setbacks, are likely to exhibit heightened negativity and vulnerability. When faced further stressful life events, they may experience more severe academic maladjustment. We will validate this in our study.

In summary, this study aims to address three critical questions: (1) Do stressful life events significantly impact academic adjustment of medical students? (2) Do depression and anxiety serve as mediators in the relationship between stressful life events and academic adjustment? (3) Are there significant differences in these mediating effects between at-risk students (those who have failed one or more exams) and other students?

## Methods

### Procedure and participants

This study was conducted in October 2022 at a university in Sichuan Province, China. Initially, we came to the university to introduce the study purpose and the targeted participants to the administrators, and secured their consent and support. They supplied a list of 320 at-risk students (those who have failed one or more exams since enrolling in college), all of whom were enrolled in the case group. To determine the number of participants in the control group, we adhered to the case-control matching criteria, maintaining a maximum ratio of 1:5 between cases and controls [24]. The control group was selected using stratified random sampling, choosing 800 students from the remaining enrolled students roughly matched to the at-risk students in terms of gender, grade, and major.

After confirming the study participants, we approached student counselors through the school administrators. The counselors then sent out invitation letters via email to the potential participants. Both the invitations and the first page of questionnaires detailed the purpose of the study and emphasized the voluntary and anonymous nature of participation. Only after the participants read and informed it, could they start to fill it out. Upon completion of the questionnaire, participants were eligible to receive a random monetary reward. Participants willing to join the study could access and submit the questionnaire through the provided website link (https://www.wjx.cn/).

Of the returned questionnaires, those completed in less than 120 s or showing logical inconsistencies (e.g., patterned responses that led to contradictory information) were excluded. A total of 1,022 valid responses were included in the final analysis, representing a response rate of 91.25%. This comprised 293 at-risk students and 729 other students; males accounted for 39.63% and females for 60.37%. Participants were distributed across various academic years and majors including clinical medicine (5 and 8 years), medical technology, and nursing. The distribution of participants was shown in Table 1.

### Measures

#### The Academic Adjustment Scale (AAS) 

It was developed by Feng et al. [12] and consists of 29 items. The scale contains five dimensions, including academic motivation (8 items), teaching model (7 items), academic ability (6 items), academic attitude (4 items), and academic environment (4 items). It utilizes a 5-point Likert scale with some reverse-scored items. Higher aggregate scores indicate better adjustment. The scale has been widely utilized among college students, with Cronbach’α coefficients exceeding 0.85 [38]. In this study, Cronbach’s alpha was 0.91.

#### The Stressful Life Events Scale (SLE)

Originally developed by Liu et al. [26] and revised by Li et al. [23], was a short and reliable assessment tool. This 16-item scale covers family, school, interpersonal, and personal stress domains. Respondents rate events on a 0 (never happened) to 5 (major impact) scale. Higher total scores indicate greater stress experienced. Cronbach’s alpha in this study was 0.85.

#### The Depression Anxiety Stress Scale (DASS-21)

Developed by Lovibond et al. [27] and revised by Gong et al. [14]. The scale exhibits stable psychometric properties and accurately reflects the levels of depression, anxiety, and stress among Chinese college students [14]. Two of the subscales were selected for this study to measure participants’ depression and anxiety, with 7 items each. Responses range from 0 (not consistent) to 3 (always consistent), with higher scores denoting more severe symptoms. Thresholds for positive depression and anxiety screening are > 10 and > 8, respectively. The Cronbach’s alpha was 0.90 for depression and 0.87 for anxiety.

### Data analysis

Data analysis involved T-tests and ANOVA to compare academic adjustment between at-risk and control groups. Pearson’s correlation assessed relationships between variables. A P-value less than 0.05 was considered statistically significant. Structural Equation Modeling (SEM) examined the mediating roles of depression and anxiety between stressful life events and academic adjustment. Acceptable model fit is indicated by a Chi-square to degrees of freedom ratio (χ²/*df*) less than 5, Comparative Fit Index (CFI), Tucker-Lewis Index (TLI), and Normed Fit Index (NFI) values exceeding 0.90, and a Root Mean Square Error of Approximation (RMSEA) below 0.08. In addition, bootstrapping was used to test the significance of mediating effects [32]. The mediating effects were considered significant if 0 was not included in the Bootstrap confidence intervals. SPSS 23.0 and AMOS 22.0 were used for data entry and analysis.

## Results

### Descriptive and correlational analysis

Significant differences on academic adjustment were observed based on residence and academic performance. Specifically, urban students demonstrated higher academic adjustment than those living in rural areas (*t* = 3.60, *p* < 0.001). At-risk students exhibited significantly lower academic adjustment than other students (*t* = 5.43, *p* < 0.001). See Table 1 for details.

**Table 1:** Demographic differences on academic adjustment

		*N* (%)	M	SD	t / F	Cohen’s d
Sex	Girls	617 (60.4%)	3.57	0.49	0.96	-
	Boys	405 (39.6%)	3.53	0.55		
Grade	Freshman	298 (29.2%)	3.63	0.54	1.66	-
	Sophomore	209 (20.5%)	3.55	0.47		
	Junior	279 (27.3%)	3.53	0.49		
	Senior	236 (23.1%)	3.51	0.55		
Major	Clinical Medicine 5-year	318 (50.6%)	3.56	0.53	1.74	-
	Clinical Medicine 8-year	98 (15.6%)	3.65	0.52		
	Medical technology	160 (25.5%)	3.48	0.51		
	Nursing	52 (8.3%)	3.58	0.42		
Residence	City	730 (71.4%)	3.60	0.53	3.60^***^	0.32
	Rural	292 (28.6%)	3.44	0.46		
Types	Other students	729 (71.3%)	3.63	0.51	5.43^***^	0.46
	At-risk students	293 (28.7%)	3.40	0.49		

***, *p* < 0.001

Exploring various dimensions of academic adjustment, at-risk students scored significantly lower in areas such as motivation, teaching model, academic ability, and attitude, along with the overall academic adjustment score (*p* < 0.05). Contrary to our expectations, at-risk students reported higher scores in the academic environment dimension (*p* = 0.002). These results detailed in Table 2.

**Table 2:** Academic adjustment for at-risk and other students

	Other students (*n* = 729)		At-risk students (*n* = 293)		t	Cohen’s d
	M	SD	M	SD		
Academic Motivation	3.54	0.67	3.07	0.65	8.24^***^	0.71
Teaching Model	3.88	0.63	3.65	0.62	4.41^***^	0.36
Academic Ability	3.78	0.60	3.49	0.59	5.52^***^	0.49
Academic Attitude	3.82	0.74	3.69	0.71	2.11^*^	0.18
Academic Environment	2.96	0.80	3.17	0.77	-3.10^**^	-0.27

***, *p* < 0.001; **, *p* < 0.01; *, *p* < 0.05

Table 3 presents descriptive statistics and the correlations among the study variables. Correlation analyses showed that stressful life events positively correlated with depression and anxiety (*p* < 0.01), while all the three variables were negatively correlated with academic adjustment (*p* < 0.01). These findings form the foundation for subsequent structural equation modeling.

Notably, at-risk students displayed higher prevalence rates of depression (46.8%) and anxiety (46.1%) compared to other students (34.0% and 40.3%, respectively). In addition, at-risk students also exhibited significantly higher incidences of major depression (22.5%) and major anxiety (30.4%) as opposed to 16.0% and 22.2% among other students.

**Table 3:** Correlations between the variables

	1	2	3	M	SD
At-risk students					
1. Stressful life events	-			10.87	7.66
2. Depression	0.42^**^			13.21	12.95
3. Anxiety	0.41^**^	0.78^**^		11.17	11.00
4. Academic adjustment	-0.28^**^	-0.59^**^	-0.52^**^	3.40	0.49
Other students					
1. Stressful life events	-			9.49	8.24
2. Depression	0.36^**^			10.45	11.48
3. Anxiety	0.37^**^	0.77^**^		9.71	10.57
4. Academic adjustment	-0.20^**^	-0.55^**^	-0.41^**^	3.63	0.51

**, *p* < 0.01

### Structural equation modeling analysis

**Fig. 1:**
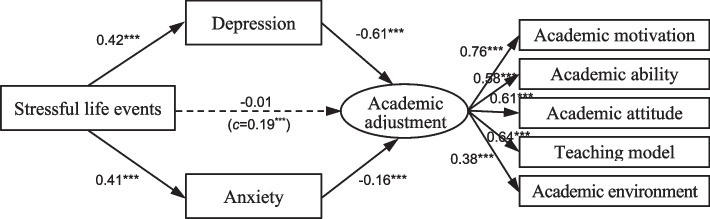
Mediating effects of depression and anxiety among at-risk students. Note.^***^, *p* < 0.001; c = total effect of independent variable on dependent variable

**Fig. 2:**
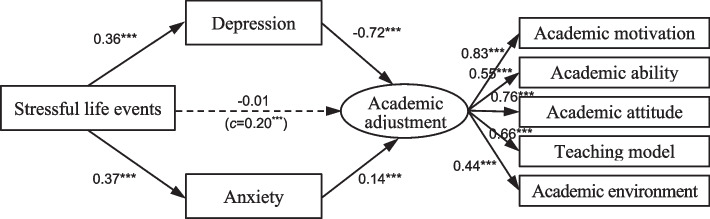
Mediating effects of depression and anxiety among other students. Note.^***^, *p* < 0.001; c = total effect of independent variable on dependent variable

Structural equation modeling was employed to investigate the relationship between stressful life events and academic adjustment, and to assess the mediating role of depression and anxiety. The SEM results for both at-risk and other students are depicted in Figs. 1 and 2, respectively. The model fit indices indicated good model fit for both groups: at-risk students (*χ*²/*df* = 2.86, NFI = 0.96, IFI = 0.98, TLI = 0.96, CFI = 0.98, RMSEA = 0.07) and other students (*χ*²/*df* = 3.04, NFI = 0.98, IFI = 0.99, TLI = 0.96, CFI = 0.99, RMSEA = 0.07). Mediating effects were evaluated using the bias-corrected percentile bootstrap method with a bootstrap size of 5000.

Among at-risk students, stressful life events had a direct negative effect on academic adjustment (*b*_1_ = 0.42, *p* < 0.001). When considering depression and anxiety as mediators, stressful life events increased both conditions (*b*_*1*_ = 0.42, *p* < 0.001; *b*_*2*_ = 0.41, *p* < 0.001), which in turn decreased academic adjustment (*b*_*1*_=-0.61, *p* < 0.001; *b*_*2*_=-0.16, *p* = 0.002). Importantly, no direct effect of stressful life events on academic adjustment was observed at this time, suggesting that depression and anxiety fully mediate the relationship.

For other students, stressful life events also had significant positive effects on depression and anxiety (*b*_*1*_ = 0.36, *p* < 0.001; *b*_*2*_ = 0.37, *p* < 0.001). There was no significant direct effect on academic adjustment when mediators were involved in the analysis. Interestingly, while depression had a significant negative effect on academic adjustment (*b*=-0.72, *p* < 0.001), but the effect of anxiety on academic adjustment was significantly positive (*b* = 0.14, *p* = 0.036).

Multiple cluster analysis was used to compare the path coefficient differences between at-risk students and the others. A significant chi-square difference (Δχ² (14) = 124.36, *p* < 0.001) and critical ratio (CR) index (depression-to-academic adjustment=-3.53; *p* < 0.05; anxiety-to-academic adjustment=-3.58, *p* < 0.05) confirmed the significant differences between the two models shown in Figs. 1 and 2.

## Discussion

### Poor academic adjustment among at-risk students

The study showed that at-risk students had significantly lower academic adjustment than other students, which was reflected in their weaker academic motivation, poorer ability and attitude, and less adjustment to the teaching model. These findings align with the research of Sang et al. [38]. In other words, students with poor academic adjustment during school are more likely to fail exams and be at risk [31]. The lack of timely intervention may worsen adolescents’ academic attitude and motivation, leading to more serious consequences [4].

It is worth noting that at-risk students scored significantly higher in the learning environment than did other students, which is inconsistent with the assumption. An analysis of the underlying reasons revealed that at-risk students tend to exaggerate the influence of the external environment (e.g., living conditions, relationships, etc.) on their studies. They may attribute their academic failure to poor learning atmosphere, or interpersonal influences, and therefore feel out of control and give up on change. It should be acknowledged that changes in college environments do affect academics [35], but more importantly, students should recognize their internal decisive role. In this regard, educators must provide timely and correct guidance.

The current results provide such suggestions for educators on developing targeted learning orientation for at-risk students at medical schools. Efforts could be made to inspire students’ own subjective role, develop their academic motivation, improve their learning ability, and adapt the university teaching mode. Self-motivation and internal attribution of learning could benefit them on avoiding academic alerts, downgrading, or even dropping out.

### Descriptive and correlation analysis of variables

Our study found that the prevalence of depression and anxiety among at-risk medical students in this study was 46.8% and 46.1%, respectively, which was not only higher than that of other students but also higher than the mean prevalence of medical students in China derived from meta-analysis [28]. It is a consensus that the mental disorders among medical students are higher than those among non-medical students and the general population in different countries worldwide [11]. The present study further identifies the most noteworthy group of medical students, at-risk medical students, about half of whom suffer from depression and anxiety. Therefore, attention should be paid not only to their academic development but also to their mental health.

Furthermore, we observed significant correlations between stressful life events and depression, anxiety, and academic adjustment. Stikkelbroek et al. [40] noted that college students who experiencing more negative life events are more likely to have depressive symptoms. This study supports and extends the findings by linking stressful life events to both depression and anxiety. Additionally, the results suggest that experiencing stressful life events is associated with poorer academic adjustment, consistent with previous research [1, 25]. These findings remind educators to focus on the mental health and academic concerns of students who experience stressful life events.

### The role of depression and anxiety in the relationship between stressful life events and academic adjustment

A key finding of the study was that stressful life events do not directly lead to academic maladjustment, with depression and anxiety playing a fully mediating role. Stress coping theory can well explain the result - stressful events do not directly lead to negative consequences, the individual’s perception and assessment of the events is critical [13]. Previous studies have also confirmed this, such as Çalışkan et al.‘s [6] study conducted in Turkey found that depression and anxiety played mediating roles in the effects of mindfulness on college adjustment; and Qiao et al. [33] noted a fully mediated role for anxiety and depressive in a study of procrastination among middle and high school students. Our study extended the subjects to medical students and found that stressful life events significantly decreased academic adjustment through increased depression and anxiety for at-risk students.

These findings consistently emphasized the importance for educators to develop targeted mental health interventions. For instance, Cognitive Behavioral Therapy (CBT) and Positive Thought Therapy have been shown to be effective in reducing students’ depression and anxiety and improving their academic performance [16].

### The different functions of anxiety between at-risk students and others

The most interesting finding of the current study was that, for students without prior exam failure, a certain level of anxiety had a positive impact on their academic adjustment. This contrasts with the results for at-risk students. Previous studies have focused mainly on the negative function of anxiety [5, 9]. However, the function was probably relied on the severity of anxiety evaluation. Wang et al. [43] found an inverted U-shaped relationship between anxiety and math performance in students with high intrinsic motivation. Similarly, among the present participants, at-risk students exhibited significantly higher incidences of major depression (22.5%) and major anxiety (30.4%) as opposed to 16.0% and 22.2% among other students. Referring to the Yerkes–Dodson Law, we assumed that an appropriate level of anxiety could promote students’ academic adjustment to some extent [44].

Thus, university educators should develop differentiated education scheme in accordance with students’ aptitude. For students without prior exam failure, proper pressure and anxiety could actually help them improve academic adjustment, while for at-risk students, decompressing and anxiety relieving works well. In addition, with reference to stress coping theory, we can hypothesize that the group differences were caused by other students possibly having more positive coping mechanisms or higher self-efficacy in the face of stressful life events [13]. Future research could address this in depth.

### Limitations

The present study is innovative not only in addressing the lack of research on influential mechanisms of medical students’ academic adjustment, but also in theoretical basis for medical school educators to develop interventions. However, there are also several limitations. First, the participants were recruited from the same university in China and may not be representative of the situation for all medical students. Ideally, multi-center and cross-national studies should be conducted. Second, the study is a cross-sectional investigation that can explain only the correlation between variables, not their causal relationship. In the future, we will continue to conduct longitudinal studies to explore the relationships between these variables. Third, the study data relied on self-reports. Although we used anonymity to try to eliminate the influence of social expectancy, recall bias may still be present. Fourth, future studies could investigate the specific coping mechanisms used by at-risk students and others, as well as the role of academic self-efficacy in mediating effects of stressful life events.

## Conclusion

At-risk medical students had worse academic adjustment. For them, experiencing stressful life events increased levels of depression and anxiety, which in turn decreased academic adjustment. However, for other students, a proper level of anxiety could benefit their academic adjustment to some extent. These findings provide a theoretical basis for developing interventions. When medical students experience stressful life events, educators should develop targeted education suggestions tailored to their characteristics to reduce the negative impact on academic adjustment.

## Data Availability

The datasets used and/or analyzed during the current study are available from the corresponding author on reasonable request.
